# *Helicobacter pylori* infection, atrophic gastritis, and disabling dementia: the Japan Public Health Center-based Prospective Study

**DOI:** 10.1265/ehpm.25-00228

**Published:** 2026-02-19

**Authors:** Takashi Matsunaga, Kazumasa Yamagishi, Hiroyasu Iso, Nobufumi Yasuda, Manami Inoue, Shoichiro Tsugane, Norie Sawada

**Affiliations:** 1 Division of Cohort Research, National Cancer Center Institution for Cancer Control, 5-1-1 Tsukiji, Chuo, Tokyo 104-0045, Japan; 2 Department of Public Health Medicine, Institute of Medicine, and Health Services Research and Development Center, University of Tsukuba, 1-1-1 Tennodai, Tsukuba, Ibaraki 305-8575, Japan; 3 Department of Public Health, Graduate School of Medicine, Juntendo University, 2-1-1 Hongo, Bunkyo-ku, Tokyo 113-8421, Japan; 4 Institute for Global Health Policy Research, National Center for Global Health and Medicine, 1-21-1 Toyama, Shinjuku, Tokyo 162-8655, Japan; 5 Department of Public Health, Kochi University Medical School, Kohasu, Okoh-cho, Nankoku, Kochi 783-8505, Japan; 6 Division of Prevention, National Cancer Center Institution for Cancer Control, 5-1-1 Tsukiji, Chuo, Tokyo 104-0045, Japan; 7 International University of Health and Welfare Graduate School of Public Health, 4-1-26 Akasaka, Minato, Tokyo 107-8402, Japan

**Keywords:** *Helicobacter pylori* infection, Atrophic gastritis, Dementia, Dietary vitamin B12 intake, Dietary folate intake

## Abstract

**Background:**

A meta-analysis reported a positive association between *Helicobacter pylori* (*H. pylori*) infection and dementia incidence. *H. pylori* infection leads to atrophic gastritis (AG) and affects the absorption of nutrients needed for nerve growth, such as vitamin B12 and folate. We aimed to clarify the associations of *H. pylori* IgG titer, AG, and their combination with disabling dementia incidence and to examine the interaction with vitamin B12 and folate.

**Methods:**

Anti-*H. pylori* immunoglobulin G (IgG) titer and pepsinogen levels were measured in 6,817 participants in 1993 (40–69 years), and the incidence of disabling dementia was followed during a median follow-up of 11.0 years. Associations of anti-*H. pylori* IgG titer, AG, and their combination with disabling dementia were examined using a multivariable-adjusted Cox proportional hazard model, with stratified analyses by dietary intake of vitamin B12 and folate.

**Results:**

1,325 (19.4%) developed disabling dementia during the follow-up period from 2006 to 2016. Among participants with low vitamin B12 intake, *H. pylori* infection and severe AG were positively associated with the risk of disabling dementia, with HRs of 1.26 (95% CI: 1.01–1.58) for *H. pylori* infection (*H. pylori* IgG titer ≥10 U/mL) and 1.34 (95% CI: 1.06–1.68) for severe AG. Additionally, having both *H. pylori* infection and AG was associated with an increased risk of dementia among individuals consuming less vitamin B12 than the median (HR 1.30, 95% CI: 1.05–1.61), compared with *H. pylori*-seronegative and AG-negative.

**Conclusions:**

The combination of *H. pylori* infection, subsequent AG, and low vitamin B12 intake may be related to increased risk of dementia.

**Supplementary information:**

The online version contains supplementary material available at https://doi.org/10.1265/ehpm.25-00228.

## 1. Introduction

The prevalence of dementia is increasing with worldwide population aging and growth. The estimated number of people with dementia was 57.4 million cases globally in 2019 and is expected to reach 152.8 million cases in 2050 [1]. Dementia can cause severe cognitive, behavioral, and physical symptoms [2] and impose a profound psychological and economic burden not only on patients but also on people around them and society [3]. Effective measures to prevent dementia are urgently needed.

Among potentially modifiable factors, infectious diseases have recently received wide attention [4–6], partly due to the neuroinflammation they cause as an underlying mechanism [7]. Among these infectious diseases, *Helicobacter pylori* (*H. pylori*) is considered to affect not only the upper digestive tract but other organs also, including the brain [8]. Indeed, a meta-analysis of five cohort and five cross-sectional studies reported a positive association between *H. pylori* and all-cause dementia [9]. If this association is causal, the prevention and treatment of *H. pylori* infection will likely have considerable potential for reducing the number of people affected by dementia.

Nevertheless, some aspects of the association between *H. pylori* infection and dementia incidence remain unclear. For example, of the six cohort [10–15] and one case-control studies [16] of the association between *H. pylori* infection and dementia reported to date, only one was conducted in an Asian population [11]. Given that the prevalence of *H. pylori* infection and lifestyle vary by population [17], the previous studies’ findings may not be applicable to Asian populations. Secondary, the association between *H. pylori* infection and dementia incidence may vary with participants’ characteristics. As a biological mechanism potentially underlying the association, atrophic gastritis (AG) caused by *H. pylori* infection may impair the absorption of vitamin B12 and folate by reducing intrinsic factor secretion and increasing intragastric pH. Deficiencies in these vitamins impair the remethylation of homocysteine to methionine, resulting in elevated homocysteine levels. High homocysteine concentrations can lead to neuronal damage through direct toxicity to neurons and endothelial cells and impaired vasodilation [9, 18–20]. Lower dietary intakes of vitamin B12 and folate may accordingly strengthen the positive association between *H. pylori* infection and dementia, but to our knowledge no previous study has examined this interaction.

Against this background, we aimed to clarify the associations of *H. pylori* infection, AG and their combination with disabling dementia incidence using Japanese population-based cohort data, including stratified analyses by dietary intake of vitamin B12 and folate.

## 2. Methods

### 2.1. Study design and participants

The Japan Public Health Center-based Prospective (JPHC) Study Cohort II is a population-based cohort study established in 1993 with subjects aged 40–69 years residing in six public health center areas. Details of the study design have been reported previously [21]. Participants reported their lifestyle and disease history and provided blood samples at the baseline survey.

**Fig. 1:**
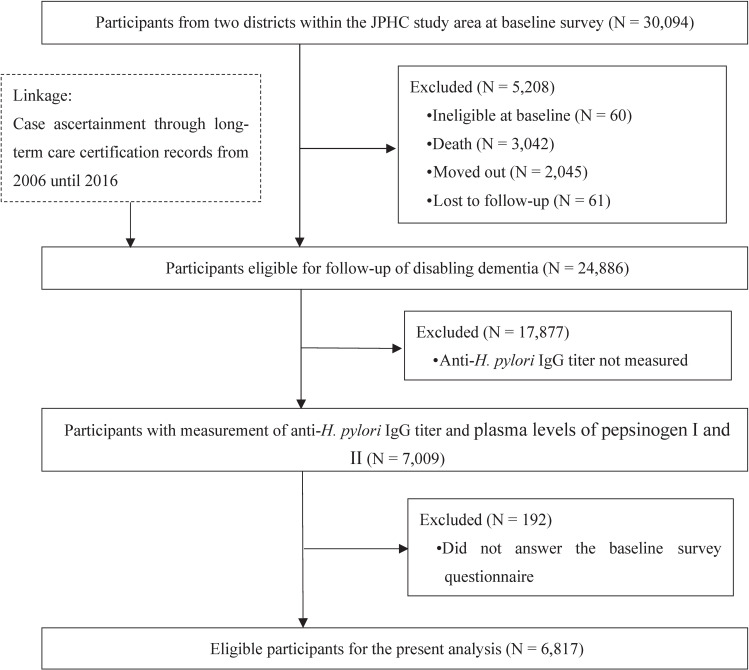
Flowchart showing the selection of eligible participants. Legend: JPHC, Japan Public Health Center-based Prospective (JPHC) Study; IgG, immunoglobulin G.

Data on the incidence of disabling dementia were obtained through national long-term care insurance (LTCI) certification records. Among the six study areas, these certification records were available in two districts (Ibaraki and Kochi areas), leaving 30,094 individuals as potential participants. The identification of disabling dementia cases started on January 1, 2006. Among the 30,094 potential participants, 5,208 became ineligible up to the start of follow-up for disabling dementia and were excluded (ineligible at baseline [N = 60], death [N = 3,042], moved out [N = 2,045], or lost to follow-up [N = 61]), leaving 24,886 individuals as eligible for follow-up for disabling dementia. For the present study, we further excluded 17,877 individuals without anti-*Helicobacter pylori* immunoglobulin (*H. pylori* IgG) titer or plasma levels of pepsinogen [PG] I and II due to their dissent against providing blood samples and 192 who did not answer the baseline survey questionnaire, leaving 6,817 individuals as eligible for the present analysis (Fig. 1).

### 2.2. Exposure

Plasma levels of *H. pylori* IgG were measured by enzyme immunoassay (E plate “Eiken” *H. pylori* Antibody II; Eiken Kagaku) and grouped into two (anti-*H. pylori* IgG titer of <10 [*H. pylori*-seronegative] and ≥10 U/mL [*H. pylori*-seropositive]) and three categories (≤3, 3.1–9.9, and ≥10 U/mL) [22, 23]. A cross-sectional study in Japan compared the diagnostic performance of the above binary categorization against endoscopic diagnosis as gold standard and reported a sensitivity of 74.8%, specificity of 99.4%, and accuracy of 96.3% [24]. Additionally, as a marker of AG, plasma levels of PG I and II were measured by latex agglutination (LZ test “Eiken” Pepsinogen I, II; Eiken Kagaku) and defined as “negative” (PG I >70 ng/mL or a ratio of PG I by PG II [PG I/II] >3.0) or “positive” (PG I ≤70 ng/mL and PG I/II ≤3.0) [22] and further as “negative” (PG I >70 ng/mL or PG I/II >3.0), “mild to moderate” (PG I ≤70 ng/mL and PG I/II ≤3.0 excluding severe cases), or “severe” (PG I ≤30 ng/mL and PG I/II ≤2.0) [25]. A meta-analysis evaluated the diagnostic performance of the above binary AG criteria against gastric endoscopic examination as gold standard and reported a sensitivity of 77%, specificity of 73%, positive predictive value of 0.77–1.25%, and negative predictive value of 99.08–99.90% [26]. To investigate a possible synergistic effect of *H. pylori* infection and AG, we also categorized participants into the following four groups: *H. pylori*-seronegative and AG-negative (*H.* *pylori*−/AG−); *H. pylori*-seropositive and AG-negative (*H. pylori*+/AG−); *H. pylori*-seronegative and AG-positive (*H. pylori*−/AG+); and *H. pylori*-seropositive and AG-positive (*H. pylori*+/AG+) [22].

### 2.3. Covariates

The present study considered the following factors as covariates: age (continuous), sex, area, smoking status, alcohol consumption, body mass index (BMI), frequency of leisure-time physical activity, living alone (as an index of social isolation), and history of diseases (cardiovascular disease [myocardial infarction and stroke], diabetes, and hypertension). These variables are considered dementia risk factors [27]. Covariates other than age, sex, and area were created based on self-reported information from the baseline questionnaire. Table 1 shows the cut-off values of categorical variables. Additionally, the present study considered dietary intakes of vitamin B12 and folate as potential factors that interact with *H. pylori* infection. These were calculated based on self-reported information from a food frequency questionnaire of the baseline survey. The validity of vitamin B12 and folate intake was assessed among subsamples (268 males and 283 females) using 28-day dietary records. The respective energy-adjusted Spearman correlation coefficients (in males and females) between intakes estimated from the questionnaire and dietary records were vitamin B12, 0.37 and 0.34; and folate, 0.39 and 0.47 (unpublished data). We categorized participants into two groups based on the medians of our sample (vitamin B12: 5.1 µg/day; folate: 271.0 µg/day). These cut-off values were generally consistent with the Dietary Reference Intakes for Japanese 2025 (vitamin B12: 4.0 µg/day; folate: 240 µg/day) [28] and the medians reported by the National Health and Nutrition Survey in Japan 2023 (vitamin B12: 4.2 µg/day; folate: 281 µg/day) [29].

**Table 1:** Characteristics of participants according to anti-*H. pylori* IgG titer.^a^

**Characteristic**	**Anti-*H. pylori* IgG titer (U/mL)**		

	**≤3**	**3.1–9.9**	**≥10**
Number of participants	1,427	560	4,830
Age (years), mean (SD)	68.0 (8.9)	70.3 (8.3)	69.9 (8.3)
Males, n (%)	348 (24.4)	164 (29.3)	1,597 (33.1)
Area, n (%)			
Ibaraki	835 (58.5)	389 (69.5)	3,315 (68.6)
Kochi	592 (41.5)	171 (30.5)	1,515 (31.4)
Occupation, n (%)			
Full-time agriculture/forestry/fishery	360 (25.2)	152 (27.1)	1,258 (26.5)
Full-time hired/self-employed/professional	374 (26.2)	123 (22.0)	1,217 (25.2)
Missing	7 (0.5)	8 (1.4)	52 (1.1)
Smoking status (pack-years), n (%)			
>30.0 pack-years	99 (6.9)	54 (9.6)	495 (10.3)
Missing	8 (0.6)	4 (0.7)	31 (0.6)
Alcohol consumption, n (%)			
≥450 g/week	101 (7.1)	37 (6.6)	355 (7.4)
Missing	1 (0.1)	3 (0.5)	16 (0.3)
Body Mass Index, n (%)			
25.0–29.9	325 (22.8)	124 (22.1)	1138 (23.6)
≥30.0	42 (2.9)	13 (2.3)	95 (2.0)
Missing	11 (0.8)	11 (2.0)	45 (0.9)
Frequency of leisure-time physical activity, n (%)			
Almost everyday	76 (5.3)	34 (6.1)	275 (5.7)
Missing	19 (1.3)	4 (0.7)	66 (1.4)
Living alone, n (%)			
Yes	37 (2.6)	16 (2.9)	136 (2.8)
Missing	10 (0.7)	4 (0.7)	33 (0.7)
History of cardiovascular disease, n (%)	36 (2.5)	20 (3.6)	109 (2.3)
History of diabetes, n (%)	68 (4.8)	46 (8.2)	248 (5.1)
History of hypertension, n (%)	294 (20.6)	123 (22.0)	1,114 (23.1)
Vitamin B12 intake (µg/day), median (IQR)	5.1 (3.5–7.1)	5.0 (3.6–7.1)	5.1 (3.6–7.1)
Folate intake (µg/day), median (IQR)	260.3 (204.8–321.4)	282.9 (213.6–342.1)	273.1 (211.2–336.8)

Notes: IQR, interquartile range.

^a^Age was as at the beginning of the follow-up for dementia incidence (January 1, 2006). Lifestyle and history of disease were examined at the same time as anti-*H. pylori* IgG titer measurement (1993).

### 2.4. Follow-up and case identification

Disabling dementia, our primary outcome, was defined according to the Japanese long-term care insurance (LTCI) certification. In the Japanese LTCI system, residents need to apply for assessment by their municipality to be eligible for LTCI benefits. This assessment requires two documents: 1) a comprehensive assessment of mental and physical conditions and 2) an opinion by a primary care physician, including a dementia rating scale. Based on these two documents, the committee of long-term care requirement certification, composed of experts in medical, health, and welfare areas, determines the care level for the applicant [30].

The dementia rating scale consists of 0, I, IIa, IIb, IIIa, IIIb, IV, and M, and are defined as follows: 0, no dementia symptoms; I, having some dementia-related symptoms but almost fully independent in daily living; IIa, sometimes having difficulty in daily living or communication outside the home but be independent in daily living life under someone’s attention; IIb, sometimes having difficulty in daily living or communication even at home but independent in daily living life under someone’s attention; IIIa, having difficulty in daily living or communication mainly during day time and requiring care; IIIb, having difficulty in daily living or communication mainly during the night and requiring care; IV, frequently having difficulties in daily living due to symptoms, behaviors, and communication problems and always requiring care; and M, having severe physical, mental, or behavioral symptoms and requiring specialized treatment [31].

The present study defined disabling dementia as a ‘certification for the level of requiring care in daily living’ and ‘dementia rating scale of IIa or above’. This definition was proved to have adequate sensitivity (73%) and specificity (96%) compared with neuropsychiatrists’ diagnoses as gold standard [32]. Participants were followed from January 1, 2006 until December 31, 2016, and the initial certification date was defined as the incidence date of the above disabling dementia.

### 2.5. Statistical analysis

The association of *H. pylori* IgG titer, AG and their combination with disabling dementia was examined with a multivariable-adjusted Cox proportional hazard model, and hazard ratios (HRs) were estimated with their 95% confidence intervals (CIs). We built one multivariable model, which incorporated covariates categorized as shown in Table 1. These models treated migration from a study area, death, loss to follow-up, and the end of follow-up (December 31, 2016) as censored events. We also conducted stratified analyses by dietary intakes of vitamin B12 and folate to examine potential interaction by these factors. These analyses stratified participants using median values of vitamin B12 and folate intakes. We also calculated *P* values for the interaction term between *H. pylori* infection (*H. pylori*− vs. *H. pylori*+) and AG (AG− vs. AG+) within each category of vitamin B12 and folate intake, followed by a likelihood ratio test. Finally, we conducted additional stratified analyses by the following factors to ascertain interactions by demographic and lifestyle variables on the association between *H. pylori* infection and dementia: sex, age, smoking status, alcohol consumption, BMI, and frequency of leisure-time physical activity. In all models, for covariates with missing values, a “missing” category was created and included as a dummy variable in the regression models along with the other categories. All statistical tests were two-sided and a *P* value <0.05 were considered to indicate statistical significance. All statistical analyses were conducted using SAS software version 9.4 (SAS Institute, Cary, NC).

## 3. Results

Among the 6,817 participants, 4,830 individuals (70.9%) had an anti-*H. pylori* IgG titer of ≥10 U/mL. Of these, 2,935 (43.1%) had AG. Table 1 shows participant characteristics by anti-*H. pylori* IgG titer. Individuals with a higher anti-*H. pylori* IgG titer tended to be male, live in the Ibaraki area, be past or current smokers, and have a history of hypertension.

During a median follow-up of 11.0 years, 1,325 participants (19.4%) had disabling dementia. Table 2 shows the association between anti-*H. pylori* IgG titer and disabling dementia. A higher anti-*H. pylori* IgG titer was not associated with the risk of disabling dementia among all participants, but was associated with increased risk among individuals consuming less vitamin B12 than the median value, with fully adjusted HRs of 1.14 (95% CI: 0.82–1.60) and 1.26 (95% CI: 1.01–1.58) for anti-*H. pylori* IgG levels of 3.1–9.9 and ≥10 U/mL, respectively, compared with ≤3 U/mL. On the other hand, anti-*H. pylori* IgG was not associated with disabling dementia regardless of folate intake.

**Table 2:** Association of anti-*H. pylori* IgG titer and disabling dementia.

**Group**	**Anti-*H. pylori* IgG titer ** **(U/mL)**	**Number of ** **participants**	**Person-years**	**Number of ** **events**	**HR (95% CI)^a^**
All participants	<10	1,987	18,506	349	Reference
	≥10	4,830	44,269	976	1.04 (0.92–1.17)
	≤3	1,427	13,494	235	Reference
	3.1–9.9	560	5,012	114	1.03 (0.82–1.30)
	≥10	4,830	44,269	976	1.05 (0.91–1.21)

Dietary vitamin B12 intake <median (5.1 µg/day)	<10	990	9,411	148	Reference
	≥10	2,419	22,355	490	1.20 (1.00–1.45)
	≤3	703	6,804	93	Reference
	3.1–9.9	287	2,607	55	1.14 (0.82–1.60)
	≥10	2,419	22,355	490	1.26 (1.01–1.58)

≥median	<10	997	9,095	201	Reference
	≥10	2,411	21,914	486	0.91 (0.77–1.08)
	≤3	724	6,690	142	Reference
	3.1–9.9	273	2,405	59	0.98 (0.72–1.34)
	≥10	2,411	21,914	486	0.91 (0.75–1.10)

Dietary folate intake <median (271.0 µg/day)	<10	1,025	9,701	175	Reference
	≥10	2,383	21,949	478	1.07 (0.90–1.28)
	≤3	773	7,418	118	Reference
	3.1–9.9	252	2,283	57	1.30 (0.94–1.79)
	≥10	2,383	21,949	478	1.16 (0.94–1.42)

≥median	<10	962	8,805	174	Reference
	≥10	2,447	22,320	498	1.01 (0.85–1.20)
	≤3	654	6,076	117	Reference
	3.1–9.9	308	2,729	57	0.86 (0.62–1.18)
	≥10	2,447	22,320	498	0.95 (0.78–1.17)

Notes: HR, hazard ratio; CI, confidence interval.

^a^Adjusted for the covariates shown in Table 1.

Table 3 shows the association between AG status and disabling dementia. Whereas having AG was not associated with the risk of disabling dementia among all participants, and regardless of folate intake, having severe AG was associated with risk among individuals consuming less vitamin B12 than the median value (HR 1.34, 95% CI: 1.06–1.68).

**Table 3:** Association of AG status and disabling dementia.

**Group**	**AG status^a^**	**Number of ** **participants**	**Person-years**	**Number of ** **events**	**HR (95% CI)^b^**
All participants	Negative	3,882	36,446	684	Reference
	Mild to moderate	2,139	19,833	412	1.07 (0.95–1.21)
	Severe	796	6,496	229	1.09 (0.94–1.27)

Dietary vitamin B12 intake <median (5.1 µg/day)	Negative	1,985	18,857	325	Reference
	Mild to moderate	1,064	9,964	202	1.13 (0.94–1.35)
	Severe	360	2,946	111	1.34 (1.06–1.68)

≥median	Negative	1,897	17,590	359	Reference
	Mild to moderate	1,075	9,869	210	1.03 (0.86–1.22)
	Severe	436	3,550	118	0.91 (0.73–1.13)

Dietary folate intake <median (271.0 µg/day)	Negative	2,020	19,138	349	Reference
	Mild to moderate	501	4,802	204	1.11 (0.93–1.33)
	Severe	887	7,710	100	1.04 (0.83–1.31)

≥median	Negative	1,862	17,309	335	Reference
	Mild to moderate	1,092	10,104	208	1.05 (0.88–1.25)
	Severe	455	3,712	129	1.18 (0.95–1.46)

Notes: AG, atrophic gastritis; HR, hazard ratio; CI, confidence interval; PG, pepsinogen.

^a^Negative: PG I >70 ng/mL or PG I/II >3.0; mild to moderate: PG I ≤70 ng/mL and PG I/II ≤3.0 excluding severe cases; severe: PG I ≤30 ng/mL and PG I/II ≤2.0.

^b^Adjusted for the covariates shown in Table 1.

Table 4 presents the association between the combination of *H. pylori* infection and AG with disabling dementia. In this analysis, having both *H. pylori* infection and AG among individuals consuming less vitamin B12 than the median was associated with an increased risk of dementia (HR 1.30, 95% CI: 1.05–1.61) compared with those who were *H. pylori*-seronegative and AG-negative, but the hazard ratio of having only *H. pylori* or AG was also higher than unity (HR 1.15, 95% CI: 0.92–1.44 for *H. pylori*-positive only; HR 1.26, 95% CI: 0.78–2.03 for AG-positive only). On the other hand, the stratified analysis by folate intake found no associations.

**Table 4:** Associations of anti-*H. pylor*i IgG titer and atrophic gastritis (AG) combinations with disabling dementia.

**Group**	**Anti-*H. pylori* IgG titer and ** **atrophic gastritis combinations^a^**	**Number of ** **participants**	**Person-years**	**Number of ** **events**	**HR (95% CI)^b^**
All participants	Anti-*H. pylori*−/AG−	1,829	17,187	310	Reference
	Anti-*H. pylori*+/AG−	2,053	19,260	374	1.01 (0.87–1.17)
	Anti-*H. pylori*−/AG+	158	1,319	39	1.13 (0.81–1.58)
	Anti-*H. pylori*+/AG+	2,777	25,009	602	1.08 (0.94–1.24)

Dietary vitamin B12 intake <median (5.1 µg/day)	Anti-*H. pylori*−/AG−	910	8,740	128	Reference
	Anti-*H. pylori*+/AG−	1,075	10,116	197	1.15 (0.92–1.44)
	Anti-*H.* *pylori*−/AG+	80	671	20	1.26 (0.78–2.03)
	Anti-*H. pylori*+/AG+	1,344	12,239	293	1.30 (1.05–1.61)

≥median	Anti-*H. pylori*−/AG−	919	8,446	182	Reference
	Anti-*H. pylori*+/AG−	978	9,144	177	0.90 (0.73–1.12)
	Anti-*H. pylori*−/AG+	78	648	19	1.04 (0.64–1.69)
	Anti-*H. pylori*+/AG+	1,433	12,770	309	0.93 (0.77–1.12)

Dietary folate intake <median (271.0 µg/day)	Anti-*H.* *pylori*−/AG−	955	9,125	156	Reference
	Anti-*H. pylori*+/AG−	1,065	10,013	193	1.07 (0.86–1.32)
	Anti-*H. pylori*−/AG+	70	576	19	1.29 (0.79–2.08)
	Anti-*H. pylori*+/AG+	1,318	11,936	285	1.12 (0.92–1.37)

≥median	Anti-*H. pylori*−/AG−	874	8,062	154	Reference
	Anti-*H. pylori*+/AG−	988	9,247	181	0.95 (0.76–1.18)
	Anti-*H. pylori*−/AG+	88	743	20	1.07 (0.67–1.72)
	Anti-*H. pylori*+/AG+	1,459	13,073	317	1.06 (0.87–1.29)

Notes: AG, atrophic gastritis; HR, hazard ratio; CI, confidence interval.

^a^Anti-*H.* *pylori*+ was defined as anti-*H. pylori* IgG titer ≥10 U/mL; and AG+ as pepsinogen I ≤70 ng/mL and pepsinogen I/II ratio ≤3.0.

^b^Adjusted for the covariates shown in Table 1.

Finally, eTable 1 shows that there were no associations between *H. pylori* infection and disabling dementia in any subgroup stratified by demographic and lifestyle variables.

## 4. Discussion

In this study of the associations of *H. pylori* infection, AG, and their combination with disabling dementia using Japanese population-based cohort data, we found that *H. pylori* infection showed no overall association with disabling dementia. However, *H. pylori* infection, AG, and their combination were associated with increased risks of disabling dementia among individuals with low dietary vitamin B12 intake. This may corroborate the biological hypothesis which implicates both AG and concurrent impaired absorption of vitamin B12 as underlying factors [9]. Based on our results, dementia incidence may be associated with not only *H. pylori* infection but also subsequent AG and low dietary vitamin B12 intake.

Among six cohort studies [10–15] and one case-control study [16] that investigated the association between *H. pylori* infection and dementia, five studies found a positive association [10, 11, 13, 15, 16]. These findings are inconsistent with our lack of association among all participants. Some factors may explain this inconsistency. First, body fatness in midlife can trigger chronic inflammation, metabolic diseases (e.g., hypertension, diabetes), and cardiovascular disease, leading to an increased risk of dementia [33]. The proportion of individuals with overweight or obesity (BMI ≥25.0 kg/m^2^) was higher in these previous studies (e.g., 40–60% [13, 16]) than in our present study (25.5%), which might account for the lack of association in the present study. Indeed, in our present stratified analyses, while *H. pylori* infection was associated with a statistically non-significant increased risk of dementia among individuals with BMI ≥25.0 kg/m^2^ (anti-*H. pylori* IgG titer ≥10 U/mL: HR 1.24 [95% CI: 0.92–1.67]), no association was seen among individuals with BMI <25.0 kg/m^2^ (HR 0.97 [95% CI: 0.82–1.14]; eTable 1). Second, differences in the number of gastric cancer deaths might account for the inconsistent findings between these previous and our present study - namely, *H. pylori* infection might lead to premature death from gastric cancer before onset of dementia. However, an additional analysis that considered gastric cancer deaths as competing events showed no material change in the subdistribution hazard ratio among all participants from the hazard ratios of the main analysis (anti-*H. pylori* IgG of 3.1–9.9 U/mL: subdistribution HR 1.02 [95% CI: 0.81–1.29]; ≥10 U/mL: subdistribution HR 1.04 [95% CI: 0.90–1.20]).

In the present study, *H. pylori* infection, AG and their combination were associated with disabling dementia only among individuals with low dietary vitamin B12 intake. Although previous studies did not examine the influence of dietary intake of vitamin B12 and folate, our results are partially consistent with the biological hypothesis that *H. pylori* infection is associated with dementia incidence [9]. According to this hypothesis, AG caused by *H. pylori* infection can hinder the absorption of vitamin B12 and folate, thereby leading to higher homocysteine concentration [9, 18]. High concentrations of homocysteine damage vascular endothelial and neuronal cells and thereby lead to brain atrophy and degeneration [34]. A meta-analysis of 10 prospective cohort studies demonstrated a positive linear dose-response relationship between homocysteine level and risk of stroke [35]. Additionally, another meta-analysis of 13 prospective cohort studies reported positive associations between homocysteine concentration and risk of all-cause dementia, Alzheimer’s disease, and vascular dementia [36]. On this basis, low dietary intakes of vitamin B12 combined with AG may exacerbate vitamin B12 deficiency developed from AG, thereby increasing the risk of dementia. Importantly, the interaction term between *H. pylori* infection and AG was non-significant in participants with low vitamin B12 intake (*P* for interaction = 0.659, data not shown in Table 4), and having AG without *H. pylor*i infection showed a non-significant but positive association with dementia (Table 4). Considering these points, dementia incidence might not necessarily depend on *H. pylori* infection but rather on AG among participants with lower vitamin B12 intakes. Interrelationships of *H. pylori* infection, AG and vitamin B12 intake in developing dementia warrant further investigations. Regarding the lack of synergistic effects of *H. pylor*i infection, AG and folate, a meta-analysis of 32 clinical studies of *H. pylori* infection and blood or urinary micronutrient levels has provided some insights, including that *H. pylori* infection and eradication influence vitamin B12 level but not folate level [37]. Accordingly, dietary folate intakes could not have interacted with *H. pylori* infection and AG on the risk of dementia in the present study.

*H. pylori* infection is thought to negatively affect cognitive function through other biological mechanisms, as well as through the impaired absorption of vitamin B12 by AG [9, 16]. We speculate about two possibilities. First, *H. pylori* can be conveyed to the brain via the oral-nasal-olfactory pathway or by circulating monocytes, possibly causing neurodegeneration [38]. Second, gut microbiota altered by *H. pylori* infection could produce amyloids and lipopolysaccharides, which may modulate signaling pathways and elevate proinflammatory cytokine levels [38].

The strengths of the present study include its prospective follow-up of disabling dementia among Japanese community-dwelling adults, identification of dementia cases through standardized methods, examination on interaction among *H. pylori* infection, AG, and dietary intakes of vitamin B12 and folate, and extensive adjustment of covariates. The following limitations should also be mentioned. First, anti-*H. pylori* IgG titer was unmeasured in a large proportion of potential participants (71.8% of potential participants residing in areas where *H. pylori* IgG measurement was conducted), which may have limited the generalizability of the findings. Second, although we used validated criteria for AG, endoscopic results were not available, which may have led to the misclassification of AG. Third, some participants may have been affected by *H. pylori* between the baseline survey and the end of follow-up, which may have brought about exposure misclassification. Against this, *H. pylori* infection is mainly acquired in childhood, and acquisition during adulthood is rare [39]. Fourth, AG acquired after the baseline survey may also have led to exposure misclassification. To our knowledge, the typical age of AG acquisition is rarely studied. Fifth, the present study could not differentiate dementia subtypes (e.g., Alzheimer’s or vascular dementia) because the present study does not collect this information. A previous analysis that used data of the JPHC Study showed no association between *H. pylori* infection and stroke [40], and thus dementia caused by the infection seemed to be mainly of the Alzheimer type. However, our relatively small number of subjects prevented an additional stratified analysis by stroke concurrent with dementia. Sixth, we did not measure the blood concentrations of vitamin B12 and folate, which forced us to rely on their dietary intakes and may have led to the misclassification of concentrations. Seventh, information on the use of vitamin B12 and folate supplements or medications was not available in our study, which may have affected the results of analyses involving these vitamins. Eighth, the present study addressed missing data by creating “missing” categories, but this may have led to the misclassification of covariates.

In conclusion, *H. pylori* infection generally was not associated with increased risk of disabling dementia among the Japanese population. Nevertheless, the combination of *H. pylori* infection, subsequent AG, and low vitamin B12 intake may be related to an increased risk of dementia. Although causality cannot be inferred from our observational data, if such a synergistic relationship is confirmed, monitoring AG and vitamin B12 status, as well as promoting adequate vitamin B12 intake, could represent promising public health strategies for dementia prevention among individuals affected by *H. pylori* infection or AG.

## Supplementary information

**Additional file 1:** **eTable 1.** Associations between anti-H. pylori IgG titer and disabling dementia stratified by demographic and lifestyle variables.

## Declarations

### Availability of data and material

The datasets supporting the conclusions of this article are available by following the instructions at: https://epi.ncc.go.jp/en/jphc/805/8155.html.
