# Supplementary material for: Helicobacter pylori infection, atrophic gastritis, and disabling dementia: the Japan Public Health Center-based Prospective Study
Source: Environ Health Prev Med. 2026 Feb 19;31:11. doi: 10.1265/ehpm.25-00228 (PMC12950342; doi:10.1265/ehpm.25-00228)
Supplement: Supplementary file 1 — Additional file 1: eTable 1. Associations between anti-H. pylori IgG titer and disabling dementia stratified by demographic and lifestyle variables. [file ehpm-31-011-s001.pdf]

Supplemental Material

Contents

1. eTable 1. Associations between anti-H. pylori IgG titer and disabling dementia stratified by demographic and lifestyle variables.

eTable 1. Associations between anti-*H. pylori* IgG titer and disabling dementia stratified by demographic and lifestyle variables.

Group	Anti-*H. pylori* IgG titer (U/mL)	Number of participants	Person-years	Number of events	HR (95% CI)[a]
Males	<10	512	4,496	92	Reference
	≥10	1,597	13,766	324	0.94 (0.74–1.20)
	≤3	348	3,228	53	Reference
	3.1–9.9	164	1,268	39	1.23 (0.80–1.88)
	≥10	1,597	13,766	324	1.02 (0.76–1.38)
Females	<10	1,475	14,010	257	Reference
	≥10	3,233	30,503	652	1.05 (0.91–1.22)
	≤3	1,079	10,266	182	Reference
	3.1–9.9	396	3,744	75	0.94 (0.72–1.23)
	≥10	3,233	30,503	652	1.03 (0.87–1.22)
≤70 years old	<10	1,054	11,095	34	Reference
	≥10	2,242	23,245	111	1.33 (0.90–1.97)
	≤3	802	8,458	22	Reference
	3.1–9.9	252	2,637	12	1.31 (0.64–2.66)
	≥10	2,242	23,245	111	1.45 (0.91–2.31)
>70 years old	<10	933	7,411	315	Reference
	≥10	2,588	21,024	865	1.00 (0.88–1.14)
	≤3	625	5,036	213	Reference
	3.1–9.9	308	2,375	102	1.01 (0.79–1.28)
	≥10	2,588	21,024	865	1.00 (0.86–1.17)
Never smoker	<10	1,520	14,461	267	Reference
	≥10	3,408	32,115	687	1.06 (0.92–1.22)
	≤3	1,126	10,781	187	Reference
	3.1–9.9	394	3,679	80	1.08 (0.83–1.40)
	≥10	3,408	32,115	687	1.08 (0.92–1.27)
Past or current smoker	<10	455	3,938	79	Reference
	≥10	1,391	11,901	283	0.96 (0.74–1.25)
	≤3	293	2,631	47	Reference
	3.1–9.9	162	1,307	32	0.85 (0.53–1.35)
	≥10	1,391	11,901	283	0.90 (0.65–1.24)

eTable 1. Continued.

Group	Anti-*H. pylori* IgG titer (U/mL)	Number of participants	Person-years	Number of events	HR (95% CI)[a]
Nondrinker	<10	1,275	11,859	251	Reference
	≥10	2,919	26,890	639	1.01 (0.87–1.17)
	≤3	920	8,641	172	Reference
	3.1–9.9	355	3,217	79	1.03 (0.79–1.35)
	≥10	2,919	26,890	639	1.02 (0.86–1.21)
Occasional or regular drinker	<10	708	6,603	98	Reference
	≥10	1,895	17,237	334	1.08 (0.86–1.36)
	≤3	506	4,842	63	Reference
	3.1–9.9	202	1,761	35	0.99 (0.64–1.51)
	≥10	1,895	17,237	334	1.07 (0.81–1.41)
BMI <25.0 kg/m^2	<10	1,461	13,540	267	Reference
	≥10	3,552	32,498	713	0.95 (0.83–1.10)
	≤3	1,049	9,837	177	Reference
	3.1–9.9	412	3,702	90	1.05 (0.81–1.36)
	≥10	3,552	32,498	713	0.97 (0.82–1.14)
≥25.0 kg/m^2	<10	504	4,795	78	Reference
	≥10	1,233	11,408	249	1.24 (0.96–1.61)
	≤3	367	3,562	56	Reference
	3.1–9.9	137	1,233	22	0.99 (0.60–1.65)
	≥10	1,233	11,408	249	1.24 (0.92–1.67)
Frequency of leisure-time physical activity Rarely	<10	1,433	13,389	247	Reference
	≥10	3,511	32,299	710	1.07 (0.92–1.24)
	≤3	1,026	9,639	173	Reference
	3.1–9.9	407	3,751	74	0.87 (0.66–1.15)
	≥10	3,511	32,299	710	1.02 (0.86–1.21)
≥1 day/month	<10	531	4,918	97	Reference
	≥10	1,253	11,438	243	0.91 (0.71–1.15)
	≤3	382	3,692	58	Reference
	3.1–9.9	149	1,226	39	1.51 (1.00–2.30)
	≥10	1,253	11,438	243	1.05 (0.78–1.41)

Notes: HR, hazard ratio; CI, confidence interval.

[a] Adjusted for the covariates shown in Table 1.
